# Association between cervical ectropion and high-grade cervical dysplasia in women of reproductive age

**DOI:** 10.1186/s12905-026-04617-6

**Published:** 2026-07-03

**Authors:** Jorge Ybaseta-Medina, Roberto Munive-Bendezú, Noemí Flores-Hernández, Luis Curotto-Palomino, Fermín Cáceres-Bellido, Víctor Barrientos-Ramos, Celia Buleje-Nuñez, Juan Panay-Centeno, Luciana Ybaseta-Soto

**Affiliations:** Facultad de Medicina Humana, Universidad Nacional San Luis Gonzaga, Av. Los Maestros s/n, Ica, 11001 Peru

**Keywords:** Cervical ectropion, Cervical dysplasia, Uterine cervical neoplasms, Human papillomavirus (HPV), Bacterial vaginosis, Sexually transmitted infections, Risk factors, Case–control study, Colposcopy

## Abstract

**Background:**

Cervical ectropion is a common anatomical condition in women of reproductive age. Although often considered a physiological variant, its potential role as a factor associated with epithelial alterations remains under debate. Exposure of immature columnar epithelium in the transformation zone may facilitate the persistence of human papillomavirus (HPV) and other infectious agents.

**Methods:**

We conducted an analytical case–control study (1:2 ratio) at a referral hospital in Ica, Peru. Cases were women with histologically confirmed high-grade cervical dysplasia (CIN2–3), while controls had consecutive negative cytology results (NILM). Cervical ectropion was assessed by standardized colposcopy. Hierarchical multivariate logistic regression was used to adjust for demographic, behavioral, and infectious factors.

**Results:**

A total of 276 women were included (92 cases and 184 controls). Cervical ectropion was observed in 33.0% of cases and in 10.3% of controls (*p* < 0.001). In multivariate analysis, ectropion remained significantly associated with high-grade cervical dysplasia (adjusted OR = 3.86; 95% CI: 1.74–8.53; *p* < 0.001). Significant associations were also found with bacterial vaginosis (adjusted OR = 2.47; 95% CI: 1.04–5.86; *p* = 0.041) and with a history of multiple sexual partners (adjusted OR = 3.55; 95% CI: 1.53–8.20; *p* = 0.003).

**Conclusions:**

Cervical ectropion was significantly associated with high-grade cervical dysplasia after adjustment for measured covariates and may constitute a potential clinical marker of epithelial vulnerability. However, residual confounding related to unmeasured HPV infection cannot be excluded. These findings support the importance of systematic screening and colposcopic surveillance, particularly in resource-limited settings where molecular HPV testing is not widely available.

**Supplementary Information:**

The online version contains supplementary material available at 10.1186/s12905-026-04617-6.

## Introduction

Cervical ectropion, also referred to as ectopy or glandular eversion, is a common anatomical condition in women of reproductive age. It consists of the exteriorization of endocervical columnar epithelium toward the exocervix, exposing it to the acidic vaginal environment and various infectious agents [1]. This exposure has been associated with chronic inflammation, mucous leukorrhea, contact bleeding, and increased susceptibility to sexually transmitted infections (STIs), including *Chlamydia trachomatis*, *Trichomonas vaginalis*, and human papillomavirus (HPV) [1–3]. Its colposcopic identification is based on the criteria established by the IARC [1].

In the transformation zone, columnar epithelium may undergo squamous metaplasia as part of the physiological maturation process [1]. This immature metaplasia should not be confused with cervical dysplasia, as it represents a physiological reparative phenomenon [2]. The coexistence of ectopy and active metaplasia may generate reactive cytological findings that complicate screening interpretation.

Several studies have suggested that ectropion may interfere with the quality of cervical cytology and be associated with squamous intraepithelial lesions, particularly in adolescents and users of hormonal contraceptives [4, 5]. Recent investigations have highlighted the relevance of cervical anatomical factors in the diagnostic accuracy of screening [6–8]. Moreover, a higher frequency of bacterial vaginosis has been reported in women with ectopy, which may influence the persistence of infectious agents [6, 7].

Cervical dysplasia is a precursor lesion of cervical cancer, and its occurrence is influenced by multiple factors, including age, sexual behavior, parity, body mass index, and STIs [8–10]. In this context, ectropion may act as an anatomical marker of epithelial vulnerability, exposing a large area of immature metaplastic cells that are susceptible to colonization by pathogens and to local inflammatory processes [3, 4, 11, 12].

However, recent systematic reviews and meta-analyses indicate that ectropion is not an independent risk factor for cervical dysplasia, with persistent HPV infection and vaginal dysbiosis identified as the main determinants of progression to high-grade lesions [11–14]. The increased susceptibility observed in young women with ectopy may be mediated by immature cervical biology and behavioral factors rather than ectropion itself [11, 12].

In Latin America, recent studies have shown that the combination of sociodemographic, gynecological, and infectious factors increases the likelihood of precancerous lesions [6, 7]. Nevertheless, evidence regarding the direct association between cervical ectropion and high-grade dysplasia (CIN 2–3) remains limited and heterogeneous, with insufficient histological confirmation and incomplete adjustment for relevant covariates [8]. Within this context, our study aims to provide additional evidence from a Latin American setting by evaluating the association between cervical ectropion and high-grade dysplasia using a case–control design with histological confirmation and multivariate adjustment, in accordance with the STROBE guidelines [15].

## Methods

### Study design

An analytical observational case–control study with a 1:2 ratio was conducted following the STROBE guidelines for epidemiological studies [15]. The primary objective was to evaluate the association between cervical ectropion and histologically confirmed high-grade cervical intraepithelial neoplasia (CIN2–3). No matching was performed, as potentially confounding variables (age, parity, BMI, sexual behavior, and sexually transmitted infections) were incorporated into a hierarchical multivariate model, thereby avoiding overmatching and allowing estimation of the adjusted association of each epidemiological domain. The methodological approach was informed by prior studies assessing risk factors for cervical pathology in resource-limited settings [13, 14].

### Population and setting

The study was conducted at Hospital Santa María del Socorro, a regional referral center for gynecology and oncology in Ica, Peru. The study period spanned from 2017 to 2019, using clinical and pathological records obtained prior to the COVID-19 pandemic. The sampling frame comprised consecutive medical records of women evaluated in the institutional colposcopy, cervical pathology, and cytological screening services.

### Eligibility criteria

#### Cases

were defined as women with histologically confirmed high-grade cervical intraepithelial neoplasia (CIN2–3) based on colposcopy-directed cervical biopsy and interpretation by certified pathologists, following standard histopathological criteria [1]. Cytology was reported using the Bethesda System; however, the case definition was based on histology (CIN grading). Histopathological confirmation of all cases reduced outcome misclassification and strengthened the internal validity of the study.

Controls were defined as women with at least two consecutive cervical cytology results negative for intraepithelial lesion or malignancy (NILM), separated by a minimum interval of six months [1]. This criterion was used to reduce outcome misclassification by increasing the likelihood of true absence of high-grade disease at the time of exposure assessment. Controls were drawn from the same source population and study period as cases (women attending the same referral hospital for cervical screening and/or diagnostic assessment). Colposcopy was performed as part of routine clinical care at the referral hospital (screening and/or diagnostic work-up), and ectropion status was abstracted from standardized colposcopy reports. At this referral hospital, colposcopy is routinely performed as part of the diagnostic work-up and/or surveillance in women referred for cervical evaluation; therefore, standardized colposcopy reports were available for both cases and controls from the same source population.

Inclusion criteria were: age between 17 and 64 years, complete clinical records, valid Pap smear results, and satisfactory colposcopy to assess exposure [1]. Exclusion criteria included prior treatment for CIN2–3 or cervical cancer, incomplete records, pregnancy, significant immunosuppression, or unsatisfactory colposcopy. Women with cytology results other than NILM (e.g., ASC-US, LSIL, HSIL, or atypical glandular cells) were not eligible as controls.

### Participant selection

A total of 310 records were initially identified from consecutive eligible medical records during the study period. After applying the exclusion criteria (*n* = 34), 276 women were included: 92 cases and 184 controls. The 1:2 ratio was used to improve statistical precision. Cases comprised consecutive eligible women with histologically confirmed CIN2–3 during the study period. After defining all eligible controls within the sampling frame, controls were selected by simple random sampling to achieve the planned 1:2 case–control ratio. The selection process is documented in a flow diagram in accordance with item 13 of the STROBE guidelines [15].

### Assessment of exposure and specimen availability

Cervical ectropion was assessed during standardized speculum examination and colposcopy; ectropion status was abstracted from the standardized colposcopy report for both cases and controls. No residual cytology or biopsy specimens were available for retrospective molecular HPV testing; therefore, HPV infection status (including high-risk genotyping and measures of persistence) could not be measured or adjusted for in this cohort.

### Variables and operational definitions

The dependent variable was histologically confirmed high-grade cervical intraepithelial neoplasia (CIN2–3). Independent variables were grouped into three domains:

Demographic and reproductive: age (< 35 vs. ≥35 years), BMI ≥ 25 kg/m², and parity ≥ 3 deliveries.

Behavioral and infectious: sexual debut < 17 years, > 3 sexual partners, history of STIs within the past 5 years, smoking, and bacterial vaginosis.

Gynecological–anatomical: presence of cervical ectropion documented by colposcopy, use of injectable contraceptives, and use of intrauterine device (IUD).

Operational definitions were based on previous literature on ectropion and cervical infection [2, 3]. Ectropion was identified from colposcopy reports following IARC criteria [1]. Bacterial vaginosis was diagnosed according to Amsel’s clinical criteria with microscopic confirmation. History of STIs was verified through clinical records and serological tests when available. Because this was a retrospective review, STIs not documented in the medical records may have been under-ascertained. HPV infection status (including high-risk HPV genotyping and measures of HPV persistence) was not available in the clinical records; therefore, adjustment for HPV as a potential confounder or mediator was not possible.

### Sample size

Sample size was calculated using Epi Info 7.2 (StatCalc), with α = 0.05, 95% confidence level, 80% statistical power, and a 2:1 ratio. A proportion of exposure among controls of 8.7% (> 3 sexual partners) and a clinically relevant OR of 2.0 were assumed, yielding a required sample size of 92 cases and 184 controls. Parameters were based on previous studies of factors associated with HPV infection and cervical dysplasia [10, 13].

### Bias control

The following measures were implemented to minimize bias:

- Selection bias: Cases comprised consecutive eligible women with histologically confirmed CIN2–3 during the study period; eligible controls were enumerated and selected by simple random sampling to achieve the planned 1:2 ratio.
- Information bias: Histopathological interpretation was performed as part of routine clinical care, prior to the present study. Pathologists were not informed of the study hypothesis, analytical objectives, or the case/control classification used for data analysis.
- Confounding: Hierarchical logistic regression was used, incorporating demographic–reproductive, behavioral–infectious, and gynecological–anatomical domains. HPV infection status (including high-risk HPV genotyping and measures of HPV persistence) was not available in the clinical records; therefore, adjustment for HPV as a potential confounder or mediator was not possible.
- Missing data: Missing values represented < 2% across all variables; double verification and sensitivity analysis excluding incomplete records were performed.

### Statistical analysis plan

Descriptive analysis: Continuous variables were expressed as means ± SD or medians [IQR] according to distribution (Shapiro–Wilk test). Categorical variables were expressed as frequencies and percentages with 95% CI.

Bivariate analysis: Pearson’s χ² test or Fisher’s exact test was used for categorical variables; Student’s t-test or Mann–Whitney U test was used for continuous variables depending on homoscedasticity (Levene’s test). Odds ratios (ORs) with 95% CI were reported.

Multivariate analysis: Variables with *p* < 0.20 in bivariate analysis entered a hierarchical binary logistic regression model, with high-grade cervical dysplasia (CIN II–III) as the dependent variable. The model was adjusted for age, BMI, multiparity, number of sexual partners, sexual debut < 17 years, STIs, bacterial vaginosis, and contraceptive type.

Goodness of fit: Hosmer–Lemeshow test (*p* > 0.05 indicating adequate fit).

Discrimination: Area under the ROC curve (AUC) with 95% CI.

Baseline balance: Standardized mean differences (SMD) were calculated; values > 0.10 were considered indicative of clinically relevant imbalance. The use of SMD allowed evaluation of group differences independently of sample size.

Software: Descriptive and bivariate analyses were performed using SPSS v26. Verification of statistical assumptions (normality, homoscedasticity, and diagnostic plots) was conducted in Jamovi 2.4. Exact confidence intervals, likelihood ratios, and the E-value were computed in Stata 17, which provides advanced tools for exact estimation and sensitivity analyses. Statistical significance was set at *p* < 0.05 (two-tailed).

### Ethical considerations

The study was reviewed and approved by the Institutional Research Ethics Committee of Hospital Santa María del Socorro (CIEI-HSMS), Ica, Peru (approval code CIEI-HSMS 2023-099).

Due to the retrospective nature of the study and the exclusive use of anonymized medical records, the Ethics Committee granted a waiver of written informed consent.

Confidentiality was ensured through data coding and anonymization of all clinical records prior to analysis.

All procedures were conducted in accordance with the principles of the Declaration of Helsinki and the recommendations of the EQUATOR Network. Ethical guidelines for research in reproductive health among vulnerable populations were also considered [14, 16].

## Results

**Fig. 1:**
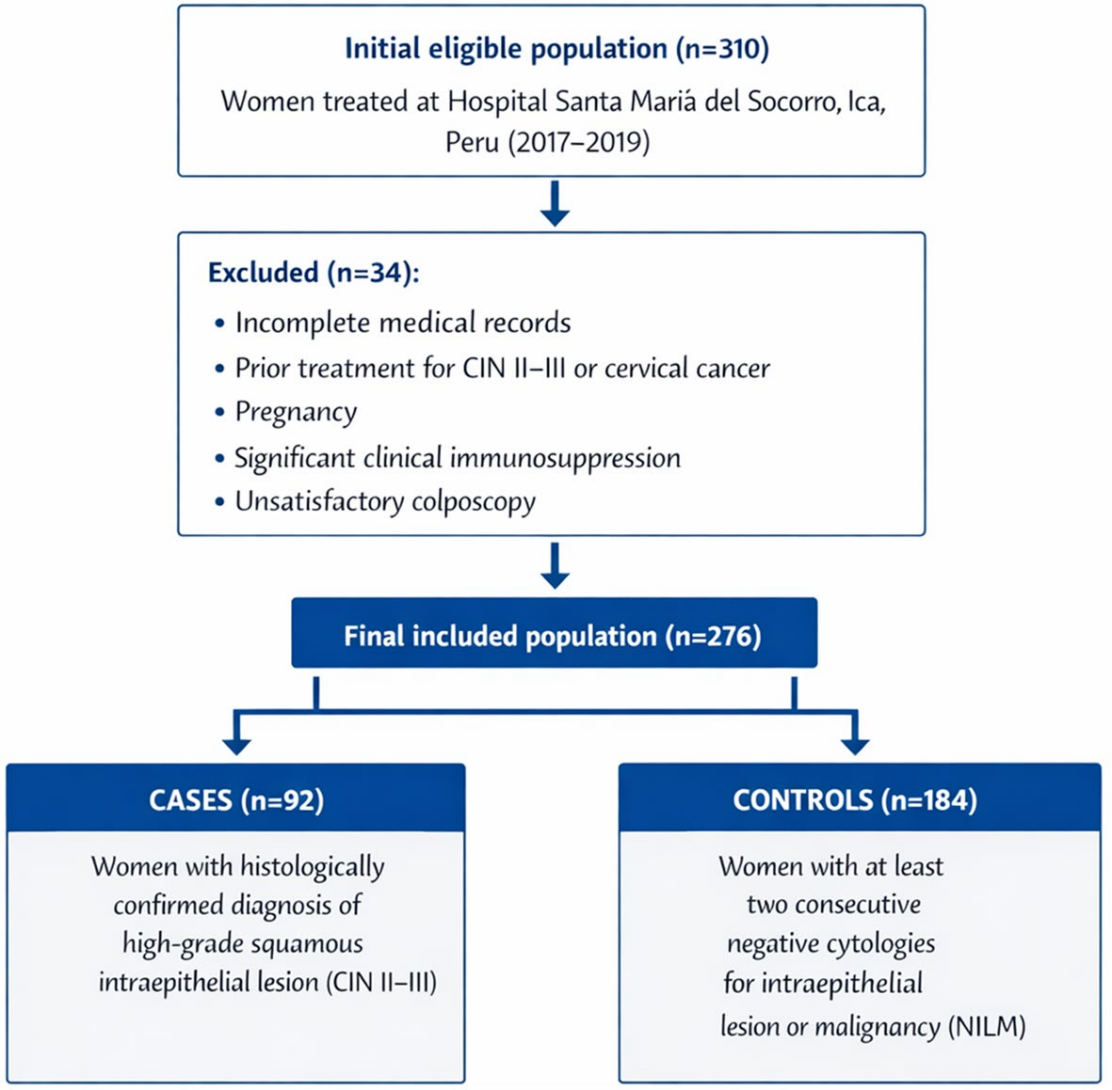
Flow diagram of participant selection in the case–control study on the association between cervical ectropion and high-grade cervical dysplasia in women of reproductive age

A total of 276 women were included in the study, of whom 92 had histologically confirmed high-grade cervical dysplasia (CIN II–III) and 184 had consecutive negative cytology results (NILM). Overall, 49 participants (17.0%) had cervical ectropion, with a significantly higher frequency among cases (33.0%) compared with controls (10.3%). The participant selection process is detailed in Fig. 1.

Baseline characteristics of the study population are presented in Table 1. Women with dysplasia were significantly younger than controls and showed higher proportions of BMI ≥ 25 kg/m², multiparity, early sexual debut, bacterial vaginosis, and multiple sexual partners. In contrast, the use of injectable contraceptives and intrauterine devices (IUDs) was more frequent among controls. Standardized mean differences (SMD > 0.10) indicated baseline imbalance across several domains, justifying the multivariate adjustment.

**Table 1:** Baseline characteristics of the study population

Variable	Controls (*n* = 184)	Cases (*n* = 92)	Total (*n* = 276)	*p*-value	SMD
Age, years (mean ± SD)	36.0 ± 8.5	29.0 ± 7.5	33.5 ± 9.2	< 0.001†	0.82
Cervical ectropion	19 (10.3%)	30 (33.0%)	49 (17.0%)	< 0.001	0.56
BMI ≥ 25 kg/m²	54 (29.3%)	45 (48.4%)	99 (35.1%)	0.007	0.27
Parity ≥ 3	55 (29.9%)	42 (45.6%)	97 (34.8%)	0.018	0.25
> 3 sexual partners	21 (11.4%)	35 (38.0%)	56 (20.3%)	< 0.001	0.61
Sexual debut < 17 years	33 (18.0%)	38 (41.3%)	70 (25.4%)	< 0.001	0.52
STIs within past 5 years	23 (12.5%)	24 (26.1%)	47 (16.9%)	0.012	0.33
Bacterial vaginosis	15 (8.2%)	29 (31.5%)	43 (15.6%)	< 0.001	0.59
Injectable contraceptive	68 (37.0%)	20 (21.7%)	88 (31.9%)	0.014	0.28
IUD	48 (26.0%)	12 (13.0%)	60 (21.7%)	0.028	0.26
Education ≤ secondary	96 (52.0%)	53 (57.6%)	149 (54.1%)	0.41	0.08
Rural residence	63 (34.0%)	35 (38.0%)	98 (35.4%)	0.52	0.06

†Student’s t-test; all other comparisons by Pearson’s χ² or Fisher’s exact test. SMD > 0.10 indicates clinically relevant imbalance

Bivariate associations between clinical, behavioral, and anatomical variables and high-grade cervical dysplasia are shown in Table 2. Elevated odds ratios were observed for age < 35 years, BMI ≥ 25 kg/m², multiparity, early sexual debut, multiple sexual partners, history of STIs, and bacterial vaginosis. Cervical ectropion showed a strong association with high-grade dysplasia. Conversely, the use of injectable contraceptives and IUDs demonstrated inverse associations.

**Table 2:** Bivariate associations between clinical, behavioral, and anatomical variables and high-grade cervical dysplasia (CIN II–III)

Domain	Variable	Cases (*n* = 92)	Controls (*n* = 184)	OR (95% CI)	*p*-value
Demographic–reproductive	Age < 35 years	61 (66.3%)	71 (38.6%)	3.10 (1.80–5.33)	< 0.001
	BMI ≥ 25 kg/m²	45 (48.4%)	54 (29.3%)	2.23 (1.25–3.95)	0.007
	Parity ≥ 3	42 (45.6%)	55 (29.9%)	1.94 (1.10–3.43)	0.018
Behavioral–infectious	> 3 sexual partners	35 (38.0%)	21 (11.4%)	4.70 (2.38–9.29)	< 0.001
	Sexual debut < 17 years	38 (41.3%)	33 (18.0%)	3.17 (1.69–5.95)	< 0.001
	STIs within past 5 years	24 (26.1%)	23 (12.5%)	2.47 (1.16–5.27)	0.012
	Current smoker	11 (11.9%)	14 (7.6%)	1.65 (0.68–4.03)	0.26
Gynecological–anatomical	Cervical ectropion	30 (33.0%)	19 (10.3%)	4.38 (2.23–8.63)	< 0.001
	Bacterial vaginosis	29 (31.5%)	15 (8.2%)	5.07 (2.30–11.2)	< 0.001
	Injectable contraceptive	20 (21.7%)	68 (37.0%)	0.46 (0.25–0.86)	0.014
	IUD	12 (13.0%)	48 (26.0%)	0.43 (0.20–0.91)	0.028

*OR* Odds ratio, *CI* Confidence interval

Pearson’s χ² or Fisher’s exact test

*p* < 0.05 indicates statistical significance

The hierarchical multivariate model (Table 3) showed that cervical ectropion remained significantly associated with high-grade cervical dysplasia (aOR = 3.86; 95% CI: 1.74–8.53; *p* < 0.001). Bacterial vaginosis and a history of multiple sexual partners also remained significantly associated. Age < 35 years continued to be significant after adjustment. The model demonstrated adequate calibration (Hosmer–Lemeshow test, *p* = 0.71) and good discriminative capacity (AUC = 0.82; 95% CI: 0.77–0.86).

**Table 3:** Hierarchical multivariate logistic regression model

Domain / Variable	aOR (95% CI)	*p*-value
Demographic–reproductive		
Age < 35 years	2.41 (1.24–4.67)	0.009
BMI ≥ 25 kg/m²	1.63 (0.84–3.17)	0.14
Parity ≥ 3	1.39 (0.73–2.65)	0.31
Behavioral–infectious		
> 3 sexual partners	3.55 (1.53–8.20)	0.003
Sexual debut < 17 years	1.97 (0.96–4.06)	0.06
STIs within past 5 years	1.74 (0.76–3.98)	0.18
Gynecological–anatomical		
Cervical ectropion	3.86 (1.74–8.53)	< 0.001
Bacterial vaginosis	2.47 (1.04–5.86)	0.041
Injectable contraceptive	0.52 (0.26–1.04)	0.07
IUD	0.61 (0.27–1.35)	0.23

Model fit: Hosmer–Lemeshow test, *p* = 0.71. Discrimination: AUC = 0.82 (95% CI: 0.77–0.86)

*aOR* adjusted odds ratio, *CI* Confidence interval. *p* < 0.05 indicates statistical significance

The proportion of missing data was < 2% across all variables, with no systematic pattern detected.

## Discussion

This study found that cervical ectropion remained significantly associated with high-grade cervical dysplasia after adjustment for measured confounding factors. Women with ectropion had significantly higher odds of CIN II–III, supporting its potential role as a clinical marker of epithelial vulnerability [3, 4]. These findings are consistent with previous investigations suggesting a relationship between ectropion and cervical abnormalities [2, 3] and add evidence through the incorporation of histological confirmation, multivariate analysis, and adherence to the STROBE guidelines [15].

From a biological perspective, exposure of immature columnar epithelium in the transformation zone may favor the persistence of infectious agents, including HPV, and facilitate local inflammatory processes that contribute to cervical carcinogenesis [11, 12]. However, recent systematic reviews have not consistently identified ectropion as a significant independent risk factor for cervical dysplasia, attributing progression to high-grade lesions primarily to persistent HPV infection and vaginal dysbiosis [6–8, 11]. These discrepancies suggest that population context, sexual behavior patterns, and interactions with other infectious factors may modulate the observed association.

In our analysis, age < 35 years remained associated with high-grade dysplasia, consistent with studies describing greater susceptibility to HPV and higher prevalence of ectropion in younger women [9, 10, 16]. Similarly, a history of multiple sexual partners showed a significant association, consistent with evidence linking sexual behavior to HPV acquisition and persistence [8, 10, 17, 18]. Bacterial vaginosis was also associated with CIN II–III, supporting the hypothesis that vaginal dysbiosis may alter the cervical microenvironment and favor viral persistence [6, 7, 14]. Although some studies have reported a possible protective effect of bacterial vaginosis against cervical cytological abnormalities [19], such differences may be explained by methodological variations, diagnostic criteria, or population characteristics.

In addition, the diagnostic performance analyses provide important insights into the potential clinical utility of cervical ectropion. Although ectropion demonstrated high specificity (89.7%) and a moderate negative predictive value, its sensitivity was low (32.6%), indicating that a substantial proportion of women with CIN2–3 would not be identified based on this finding alone. Therefore, cervical ectropion should not be considered a screening tool for high-grade cervical dysplasia. However, in women with absent, incomplete, or uncertain screening histories, the presence of ectropion during routine gynecological examination may serve as a clinical indicator prompting cervical cytology, HPV testing, or closer colposcopic evaluation. Consequently, ectropion may have value as a complementary clinical marker for risk stratification rather than as a standalone screening method.

Regarding contraceptive methods, the use of injectable contraceptives and intrauterine devices (IUDs) showed inverse associations, although these did not reach statistical significance. The literature suggests that IUDs may induce local immunological responses that reduce the likelihood of cervical progression [20, 21], whereas evidence on hormonal contraceptives remains heterogeneous and dependent on type, duration of use, and study population [22, 23].

### Strengths and limitations

Key strengths include the use of a hierarchical model to evaluate differentiated epidemiological domains, histological confirmation of all cases, and standardized colposcopic assessment of ectropion, which may reduce the risk of misclassification bias.

### Limitations

The case–control design precludes establishing temporality or causality. Molecular HPV testing was not available in the study setting, and no residual cytology or biopsy specimens were available for retrospective molecular analysis; therefore, infection and persistence of high-risk HPV—major determinants of CIN—could not be measured or adjusted for, and this omission may have confounded the observed association between ectropion and CIN2–3. HPV may also lie on the causal pathway (ectropion → HPV acquisition/persistence → CIN), meaning that the observed association could be partially mediated by unmeasured HPV persistence.

To assess the potential impact of unmeasured HPV-related confounding, we performed quantitative sensitivity analyses (Supplementary Material), including a 2 × 2 diagnostic table for ectropion (sensitivity, specificity, PPV, NPV), scenario analyses modeling plausible cytology false-negative rates (10% and 20%), and an E-value calculation for the adjusted odds ratio. These analyses indicated low sensitivity but high specificity for ectropion and suggested that an unmeasured confounder would require a strong association with both ectropion and CIN2–3 to fully explain the observed association; nevertheless, residual confounding by HPV cannot be definitively excluded.

Additional limitations include the lack of information regarding oral contraceptive exposure, laboratory-confirmed *Chlamydia trachomatis* infection, and partner circumcision status, which may also have contributed to residual confounding. The hospital-based sample may limit generalizability to the broader community, as reported in other observational studies conducted in similar clinical settings [24].

#### Practical note

See Supplementary Material for full details of the sensitivity analyses and the E-value calculation.

### Clinical implications and future research

Our findings suggest that the presence of ectropion may help stratify risk during cervical screening. Women with persistent ectropion—especially when combined with other risk factors such as young age, multiple sexual partners, or bacterial vaginosis—may warrant closer follow-up and prompt colposcopic evaluation in accordance with current cervical screening recommendations [25, 26]. A more individualized surveillance strategy may also help avoid unnecessary invasive interventions associated with adverse reproductive and perinatal outcomes [27].

Timely diagnosis and treatment of bacterial vaginosis in women with ectropion could be explored as a potential preventive strategy, although randomized clinical trials are needed to test this hypothesis. Future research priorities include multicenter longitudinal studies incorporating molecular HPV typing, characterization of the vaginal microbiota, and assessment of local immune responses to clarify the mechanisms linking ectropion and cervical carcinogenesis.

It would also be valuable to evaluate whether interventions targeting ectropion (e.g., cauterization or cryotherapy) modify the risk of progression of cervical lesions in high-risk populations [28].

## Conclusions

This case–control study found a significant association between cervical ectropion and high-grade cervical dysplasia after adjustment for measured clinical, behavioral, and infectious factors. Our findings suggest that anatomical characteristics such as ectropion may contribute to cervical vulnerability and support considering ectropion within a comprehensive risk assessment, particularly in young women with multiple associated factors.

Given that the study design does not allow causal inference and that HPV infection status could not be measured, prospective investigations incorporating molecular HPV testing are required to further evaluate these associations and to assess potential preventive interventions in high-risk settings. 

## Supplementary Information

Supplementary Material 1.

## Data Availability

The datasets generated and/or analyzed during the current study are available from the corresponding author upon reasonable request.
