# Supplementary material for: Association between cervical ectropion and high-grade cervical dysplasia in women of reproductive age
Source: BMC Womens Health. 2026 Jul 3;26:352. doi: 10.1186/s12905-026-04617-6 (PMC13359557; doi:10.1186/s12905-026-04617-6)
Supplement: Supplementary file 1 — Supplementary Material 1. [file 12905_2026_4617_MOESM1_ESM.docx]

Supplementary Methods — Sensitivity analyses and E‑value calculation. Includes formulas, a worked numerical example using the 2×2 counts, and Supplementary Table 1 (diagnostic performance of cervical ectropion for CIN2–3).

**Supplementary Table 1. Diagnostic performance of cervical ectropion for CIN2–3**

| **Measure** | **Value** | **95% CI** |
| --- | --- | --- |
| Cases with ectropion | 30 | — |
| Cases without ectropion | 62 | — |
| Controls with ectropion | 19 | — |
| Controls without ectropion | 165 | — |
| Sensitivity | 32.6% | 23.3%–43.1% |
| Specificity | 89.7% | 84.2%–93.6% |
| PPV | 61.2% | 47.8%–73.2% |
| NPV | 72.7% | 66.9%–78.0% |
| LR+ | 3.14 | 2.05–4.81 |
| LR− | 0.75 | 0.64–0.88 |
| aOR (multivariate) | 3.86 | 1.74–8.53 |
| E‑value (aOR) | 7.18 | — |
| E‑value (lower 95% CI) | 2.88 | — |

***Note.*** Values calculated from the 2×2 contingency table; exact 95% CIs by Clopper–Pearson; LR CIs by log‑transformation (Simel et al.). See Supplementary Methods for calculation details, the 10% and 20% false‑negative scenarios, and the Excel workbook with worked examples.

Supplementary Methods — Sensitivity analyses and E‑value calculation

1. Diagnostic performance calculations Sensitivity, specificity, positive predictive value (PPV) and negative predictive value (NPV) were calculated from the 2×2 contingency table using standard definitions:

- Sensitivity = TP / (TP + FN)
- Specificity = TN / (TN + FP)
- PPV = TP / (TP + FP)
- NPV = TN / (TN + FN)

2. Exact 95% confidence intervals Exact binomial 95% confidence intervals for sensitivity, specificity, PPV and NPV were computed using the Clopper–Pearson method [17].

3. Likelihood ratios and their confidence intervals Positive and negative likelihood ratios were calculated as:

- $\mathrm{LR}+=\frac{\text{sensitivity}}{1-\text{specificity}}$
- $\mathrm{LR}-=\frac{1-\text{sensitivity}}{\text{specificity}}$

Confidence intervals for LR+ and LR− were estimated on the log scale using the method described by Simel et al.: compute the standard error of $\ln(\mathrm{LR})$, derive the CI on the log scale, then exponentiate back to the original scale [18].

4. Scenario reclassification for cytology false negatives to assess the potential impact of nondifferential misclassification among controls due to false‑negative cytology, we implemented two conservative scenarios:

- Scenario A (10% false negatives): Reclassify 10% of controls as cases. Operationally, randomly select (or select deterministically by proportion from the control counts) the corresponding number of controls with ectropion and without ectropion in proportion to their observed distribution, then recompute the 2×2 counts and the crude OR.
- Scenario B (20% false negatives): Same procedure using 20% reclassification.

For transparency, report the recalculated crude ORs for each scenario and compare them with the observed crude and adjusted ORs. State whether the direction and magnitude of association are materially altered.

5. E‑value calculation We computed the E‑value following VanderWeele and Ding (2017) [19]:

$$\text{E‑value}=RR+\sqrt{RR\cdot(RR-1)}$$

When the outcome is rare, the adjusted odds ratio (aOR) can be used as an approximation of the risk ratio (RR) for sensitivity purposes; we therefore report the E‑value for the point estimate (using aOR) and for the lower bound of the 95% CI. Provide both values and interpret them: the E‑value quantifies the minimum strength of association (on the risk ratio scale) that an unmeasured confounder would need to have with both exposure and outcome, conditional on measured covariates, to explain away the observed association.

6. Worked numerical example (one short example to ensure reproducibility) Using the 2×2 counts in Supplementary Table 1 (TP = 30; FN = 62; FP = 19; TN = 165):

- Sensitivity = $30/(30+62)=0.326$→ 32.6% (Clopper–Pearson 95% CI: 23.3%–43.1%).
- Specificity = $165/(165+19)=0.897$→ 89.7% (Clopper–Pearson 95% CI: 84.2%–93.6%).
- LR+ = $0.326/(1-0.897)=0.326/0.103=3.16$(rounded in table to 3.14). Compute $\ln(\mathrm{LR}+)$and its SE to derive the CI on the log scale, then exponentiate.
- E‑value (using aOR = 3.86):

$$\text{E‑}\text{value}=3.86+\sqrt{3.86\cdot(3.86-1)}\approx7.18$$

E‑value for lower 95% CI (1.74): compute using RR = 1.74 → E‑value ≈ 2.88.

Include the intermediate arithmetic in the Supplementary Methods so reviewers can follow each step.

**7. Software and reproducibility statement**

Primary statistical analyses were conducted using SPSS v26 and Jamovi 2.4, as described in the main Statistical Analysis Plan. Exact binomial confidence intervals, likelihood ratios, and E-value calculations were performed in Stata 17 using exact estimation procedures and sensitivity analysis tools. All calculations presented in these Supplementary Methods can be reproduced using the formulas and procedures described above.

**8. Supplementary files**

The supplementary material provided with this manuscript includes:

- *Supplementary_Material_Sensitivity_Analyses.docx*: Supplementary Methods and Supplementary Table 1 containing diagnostic performance metrics, sensitivity analyses, likelihood ratios, and E-value calculations.
- *(Optional)* *Supplementary_Table1_2x2_and_metrics.xlsx*: Spreadsheet containing the formulas and calculations used for diagnostic and sensitivity analyses.
